# An rRNA fragment in extracellular vesicles secreted by human airway epithelial cells increases the fluoroquinolone sensitivity of *P. aeruginosa*

**DOI:** 10.1152/ajplung.00150.2022

**Published:** 2023-05-31

**Authors:** Katja Koeppen, Thomas H. Hampton, Roxanna Barnaby, Carolyn Roche, Scott A. Gerber, Young Ah Goo, Byoung-Kyu Cho, Danielle M. Vermilyea, Deborah A. Hogan, Bruce A. Stanton

**Affiliations:** ^1^Department of Microbiology and Immunology, Geisel School of Medicine at Dartmouth, Hanover, New Hampshire, United States; ^2^Norris Cotton Cancer Center, Geisel School of Medicine at Dartmouth, Lebanon, New Hampshire, United States; ^3^Mass Spectrometry Technology Access Center, McDonnell Genome Institute, Washington University School of Medicine, St. Louis, Missouri, United States

**Keywords:** antibiotic resistance, ciprofloxacin, EVs, host-pathogen interaction, Pseudomonas

## Abstract

Lung infections caused by antibiotic-resistant strains of *Pseudomonas aeruginosa* are difficult to eradicate in immunocompromised hosts such as those with cystic fibrosis. We previously demonstrated that extracellular vesicles (EVs) secreted by primary human airway epithelial cells (AECs) deliver microRNA let-7b-5p to *P. aeruginosa* to suppress biofilm formation and increase sensitivity to beta-lactam antibiotics. In this study, we show that EVs secreted by AECs transfer multiple distinct short RNA fragments to *P. aeruginosa* that are predicted to target the three subunits of the fluoroquinolone efflux pump MexHI-OpmD, thus increasing antibiotic sensitivity. Exposure of *P. aeruginosa* to EVs resulted in a significant reduction in the protein levels of MexH (−48%), MexI (−50%), and OpmD (−35%). Moreover, EVs reduced planktonic growth of *P. aeruginosa* in the presence of the fluoroquinolone antibiotic ciprofloxacin by 20%. A *mexGHI-opmD* deletion mutant of *P. aeruginosa* phenocopied this increased sensitivity to ciprofloxacin. Finally, we found that a fragment of an 18S ribosomal RNA (rRNA) external transcribed spacer that was transferred to *P. aeruginosa* by EVs reduced planktonic growth of *P. aeruginosa* in the presence of ciprofloxacin, reduced the minimum inhibitory concentration of *P. aeruginosa* for ciprofloxacin by over 50%, and significantly reduced protein levels of both MexH and OpmD. In conclusion, an rRNA fragment secreted by AECs in EVs that targets the fluoroquinolone efflux pump MexHI-OpmD downregulated these proteins and increased the ciprofloxacin sensitivity of *P. aeruginosa*. A combination of rRNA fragments and ciprofloxacin packaged in nanoparticles or EVs may benefit patients with ciprofloxacin-resistant *P. aeruginosa* infections.

**NEW & NOTEWORTHY** Human RNA fragments transported in extracellular vesicles interfere with *Pseudomonas aeruginosa* drug efflux pumps. A combination of rRNA fragments and ciprofloxacin packaged in nanoparticles or EVs may benefit patients with antibiotic-resistant *P. aeruginosa* infections.

## Graphical abstract

**Figure:**
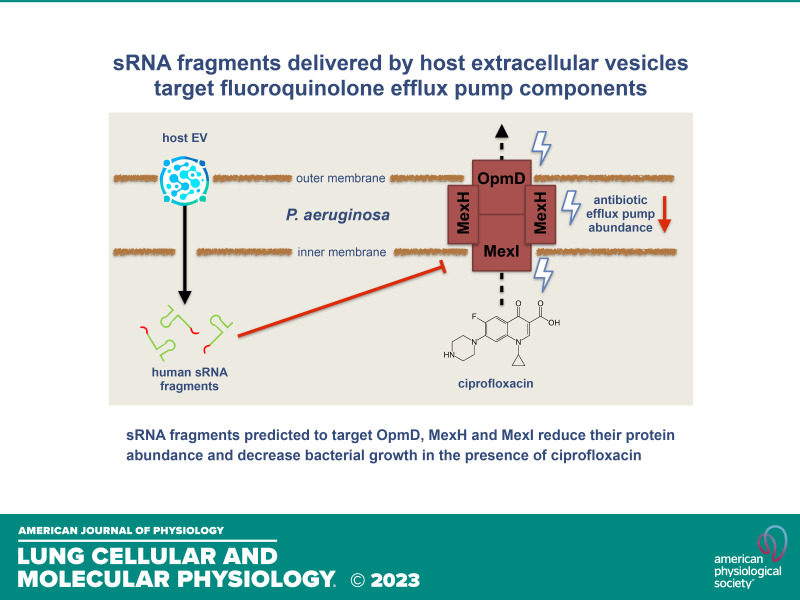

## INTRODUCTION

*Pseudomonas aeruginosa* is an opportunistic bacterial pathogen that can cause chronic infections in immunocompromised hosts, leading to increased morbidity and mortality in individuals with cystic fibrosis (CF), chronic obstructive pulmonary disease, ventilator-associated pneumonia, and burn wounds (1). *P. aeruginosa* is also emerging as a pathogen that commonly coinfects and exacerbates COVID-19 infections (2–5). The rise of difficult-to-eradicate, antibiotic-resistant strains of *P. aeruginosa* is posing treatment challenges and increasingly becoming a concern in healthcare settings (6–9). Multidrug-resistant strains of *P. aeruginosa* are estimated to have caused ∼32,600 hospital-acquired infections and 2,700 deaths in the United States in 2017 and have been rated as a serious threat by the Centers for Disease Control and Prevention in a 2022 report (10). Thus new approaches are needed to eradicate antibiotic-resistant infections by *P. aeruginosa.*

Bidirectional host-pathogen communication through short RNA (sRNA) in extracellular vesicles (EVs) and the resulting regulation of the host immune response as well as bacterial phenotypes such as antibiotic sensitivity have become a research focus in recent years (11–19). We have previously shown that *P. aeruginosa* outer membrane vesicles deliver a transfer RNA (tRNA) fragment to human airway epithelial cells (AECs), suppressing the host innate immune response to infection, thereby facilitating chronic infections (11). Eukaryotic EVs have also been reported to contain tRNA fragments (20–22), and tRNA fragments regulate gene expression in eukaryotes and prokaryotes (23–27) and play a role in various human diseases (28, 29). Previous studies have primarily focused on the regulatory effects of tRNA fragments from a given organism on that same organism, rather than on interspecies cross talk. Much less is known about the role of tRNA fragments and other sRNAs in interkingdom signaling between eukaryotic and prokaryotic organisms.

Besides tRNA fragments and messenger RNAs (mRNAs), eukaryotic EVs contain a diverse assortment of noncoding RNA species, including long noncoding RNAs (lncRNAs), microRNAs (miRNA), piwi-interacting RNAs (piRNAs), Y RNAs, antisense RNAs, transfer RNAs (tRNAs), tRNA fragments, and ribosomal RNAs (rRNAs) (20, 22, 30–33). EVs as well as their miRNA cargo are involved in immune regulation, and their secretion and RNA content have been shown to be dysregulated in patients with airway diseases (34–38). For example, a recent study found that increased levels of miR-223-3p or miR-451a and decreased levels of miR-27b-3p in sputum supernatants correlated with exacerbation in children with CF and infection with *Aspergillus*, *Haemophilus*, or *Pseudomonas*, respectively, suggesting the involvement of EV miRNAs in pulmonary exacerbation (39). Two recent studies provide a comprehensive characterization of the sRNA content of EVs secreted by AECs: one reporting changes in the sRNA content of EVs secreted by the human airway epithelial cell line A549 in response to viral infection (40) and another describing cigarette smoke-induced changes in the RNA content of EVs secreted by primary small airway epithelial cells (41). Prior studies of EVs secreted by AECs have focused mainly on their miRNA cargo (19, 42–44), whereas the function of other sRNA species contained in AEC EVs has not yet been reported. While there is a growing body of literature on the regulatory effects of EV sRNAs within a given organism, little is known about the ability of sRNAs secreted in eukaryotic EVs to regulate gene expression and function in prokaryotes. For example, eukaryotic miRNAs affect the growth of gut microbes through an unknown mechanism (45–47). Recently, we provided the first direct evidence that a miRNA, let-7b-5p, in EVs secreted by human airway epithelial cells is transferred to *P. aeruginosa*, where it increases beta-lactam antibiotic sensitivity and decreases biofilm formation by reducing the abundance of the beta-lactamase AmpC and several proteins required for biofilm formation (19). Here, we report that human EVs also reduce the abundance of all three subunits of the fluoroquinolone efflux pump MexHI-OpmD and increase fluoroquinolone sensitivity, an effect mediated by an rRNA fragment secreted in EVs.

## METHODS

### Culture of Airway Epithelial Cells and EV Isolation

Deidentified primary human AECs were obtained from Dr. Scott Randell (University of North Carolina, Chapel Hill, NC) and cultured in BronchiaLife basal medium (Lifeline Cell Technology, Frederick, MD; cat. no. LM-0007) supplemented with the BronchiaLife B/T LifeFactors Kit (Lifeline Cell Technology; cat. no. LS-1047) as well as 10,000 U/ml penicillin and 10,000 μg/mL streptomycin (Sigma-Aldrich, St. Louis, MO; cat. no. P4333), as previously described (48). EVs were isolated from AEC culture supernatants (passages 4–8) with the ExoQuick-TC EV isolation kit (System Biosciences, Palo Alto, CA; cat. no. EXOTC50A-1) and characterized by Nanoparticle Tracking Analysis and Western blots, as previously reported (49).

### Bacterial Strains and Culture

For all experiments, *P. aeruginosa* strains PA14 and PAO1 and the mucoid CF clinical isolate SMC1585 (50) were cultured in Luria broth (LB; Thermo Fisher Scientific, Waltham, MA). A deletion mutant for the fluoroquinolone efflux pump *mexGHI-*opmD in the PAO1 background (strain DW101, PAO1ΔGHID) and the matching parental PAO1 wild-type strain were generously provided by Dr. Helen Zgurskaya from the University of Oklahoma (51). Deletion of *opmD* in the PAO1*ΔmexGHI-*opmD strain was confirmed by PCR. Briefly, RNA was isolated from PAO1*ΔmexGHI-opmD* and the parental PAO1 wild-type strain using the miRNeasy Mini Kit (Qiagen, Germantown, MD; cat. no. 217004), cDNA was synthesized with the Invitrogen RETROscript Reverse Transcription Kit (Thermo Fisher Scientific; cat. no. 10585595), and the presence of *opmD* was interrogated by PCR amplification using the following primers: forward 5′- CCTGGTGGAGTTTCTTCGAC-3′ and reverse 5′- CGTCGTAGTCCAGCTGTTGT-3′.

*P. aeruginosa* strains expressing either the 18S rRNA spacer fragment under the control of an arabinose-inducible promoter or the empty vector control were produced by transforming PA14 with a pMQ70 expression vector (52) containing either the rRNA fragment sequence (listed in Table 3) as an insert or no insert (control). Strains were cultured with 300 µg/ml carbenicillin (Sigma-Aldrich; cat. no. C1389) to select for bacteria containing the plasmid, and 100 mM l-(+)-arabinose (Sigma-Aldrich; cat. no. A3256) were added during experiments to induce expression of the rRNA fragment.

### Bacterial Growth Curves and CFU Count

The minimal inhibitory concentration (MIC) of the fluoroquinolone antibiotic ciprofloxacin was determined by incubation of each *P. aeruginosa* strain with eight different concentrations of ciprofloxacin (0–0.25 µg/ml) in triplicate for 20 h in a plate reader at 37°C. The optical density at 600 nm (OD_600_) was recorded every 15 min, and the MIC for ciprofloxacin was calculated by fitting the growth curve data with a Gompertz function, as previously described (53, 54). At the beginning of the 20-h growth curves, 5,000 colony-forming units (CFUs) of *P. aeruginosa* were exposed to 1.5 × 10^9^ EV/ml, corresponding to a concentration of EVs measured in AEC culture supernatants by us, as well as in human bronchoalveolar lavage fluid (49, 55). During exposure of *P. aeruginosa* to EVs, ciprofloxacin (Sigma-Aldrich; cat. no. 17850-5 G-F) was present in the culture medium at a concentration equivalent to one-third to one-half the MIC of a given strain, as indicated in the figure legends. CFUs per milliliter for viable planktonic bacteria were obtained by dilution plating of supernatants.

### Proteomics of *P. aeruginosa*

CFUs (1.5 × 10^7^) of PA14 were exposed to 1.5 × 10^10^ EV secreted by AEC from three donors or vehicle controls for a PA14:EV ratio of 1:1,000 and incubated for 15 h at 37°C at 225 rpm. Samples were processed and tandem mass tag (TMT) labeled as described previously (19). All six samples were run in duplicate, and peak intensities from duplicate runs were averaged and filtered to retain 1,619 proteins that were detected in all replicate samples. Differentially abundant proteins were identified using QPROT (56). The mass spectrometry proteomics data have been deposited to the ProteomeXchange Consortium (57) via the MassIVE (58) partner repository with the data set identifier PXD033213.

In addition, following overnight cultures with 300 µg/ml carbenicillin to select for bacteria with the plasmid containing the rRNA fragment or the empty plasmid, three different clones of the PA14 strains expressing either the rRNA fragment or the empty vector control were cultured with 100 mM arabinose for 15 h at 37°C at 225 rpm to induce expression of the rRNA fragment. Cell pellets were washed with PBS and lysed, and proteins (200 µg of each sample) were purified by acetone/trichloroacetic acid precipitation overnight at −20°C. After the pellet was washed with ice-cold acetone, the resulting protein pellet was resuspended in 50 μL of 8 M urea in 400 mM ammonium bicarbonate, pH 7.8, and reduced with 4 mM dithiothreitol at 50°C for 30 min, and cysteines were alkylated with 18 mM iodoacetamide for 30 min. The solution was then diluted to <2 M urea, and trypsin (Promega, Madison, WI) was added at a final trypsin/protein ratio of 1:100 before overnight incubation at 37°C. The resulting peptides were desalted using solid-phase extraction on a C18 spin column and eluted in 240 μL of 80% acetonitrile in 0.1% formic acid. All samples were analyzed in technical duplicates by liquid chromatography-tandem mass spectrometry (LC-MS/MS) using a nanoElute coupled to a timsTOF Pro Mass Spectrometer (Bruker Daltonics). Two hundred nanograms of each digested peptide sample were loaded on a capillary C18 column (25 cm long, 75-μm inner diameter, 1.6-μm particle size, and 120-Å pore size; IonOpticks). The flow rate was kept at 300 nL/min. *Solvent A* was 0.1% formic acid (FA) in water, and *solvent B* was 0.1% FA in acetonitrile (ACN). The peptides were separated on a 100 min analytical gradient from 2% ACN/0.1% FA to 35% ACN/0.1% FA for a total of 120 min gradient. The timsTOF Pro was operated in the PASEF mode. Mass spectrometry (MS) and tandem mass spectrometry (MS/MS) spectra were acquired from 100–1,700 *m*/*z*. The inverse reduced ion mobility 1/K_0_ was set to 0.60−1.60 V·s/cm^2^ over a ramp time of 100 ms. Data-dependent acquisition was performed using 10 parallel accumulation-serial fragmentation (PASEF) MS/MS scans per cycle with a near 100% duty cycle. The resulting protein tandem MS data were queried for protein identification and label-free quantification against the Uniprot *Pseudomonas aeruginosa* strain UCBPP-PA14 database (proteome ID UP000000653, retrieved on Feb 2, 2022) using MaxQuant v2.0.3.1. The following modifications were set as search parameters: peptide mass tolerance at 20 ppm, trypsin digestion cleavage after K or R (except when followed by P), two allowed missed cleavage sites, carbamidomethylated cysteine (static modification), and oxidized methionine, deaminated asparagine/glutamine, and protein N-term acetylation (variable modification). Search results were validated with peptide and protein false discovery rate both at 0.01. Label-free quantitation intensities were averaged across technical replicates, filtered to retain 1,911 proteins detected in at least two replicate samples, and normalized before QPROT analysis (56). The mass spectrometry proteomics data have been deposited to the ProteomeXchange Consortium (57) via the PRIDE (59) partner repository with the data set identifier PXD033165.

### Characterization of EV sRNA Content

Previously, we characterized the miRNA content of AEC EVs (19); here, we focus on the EV sRNA content beyond miRNAs. The raw data as well as count tables of aligned reads provided by System Biosciences can be accessed through NCBI’s Gene Expression Omnibus (GEO Series Accession No. GSE174690). In addition to the alignment-based characterization, we also performed a sequence-based characterization of EV sRNA content. Adaptor trimming of raw reads was performed with fastp version 0.20.0 (60). Trimmed reads from EVs secreted by three donors of AECs yielded close to 500,000 unique sequences, ∼40,000 of which were detected in all 3 biological replicates. Count tables with the number of reads per sample for each unique sequence were generated using the Unix command “cat fastp.fastq.gz | gunzip | awk '(NR%4==2)' | sort | uniq -c | sort -k 1 –n.” To determine the origin of the most abundant unique EV sRNA sequences, we ran command line BLAST+ (61) for 1346 sequences that had a minimum of 100 counts per sample and a length of at least 20 nucleotides (nt). Arguments to the blastn function included -taxids 9606 (homo sapiens), -perc_identity 100 (requiring a perfect match), -qcov_hsp_perc 100 (requiring 100% query coverage), and -task blastn-short (to adjust for a short input sequence). GenBank accessions returned by BLAST were annotated using the R package rentrez (62). Subsequently, RNA sequencing (RNA-seq) samples from EV-exposed *P. aeruginosa* were used to assess whether human sRNAs are transferred to *P. aeruginosa* by EVs.

### Characterization of sRNA Transferred to *P. aeruginosa* and Transcriptional Effects

Existing RNA-seq data of *P. aeruginosa* exposed to PBS vehicle control in triplicate or to EVs from three AEC donors (19) were reanalyzed to identify EV-mediated transfer of sRNA other than miRNAs to *P. aeruginosa.* Raw RNA-seq data for sRNA can be accessed through NCBI's Gene Expression Omnibus (GEO Series Accession No. GSE174710). For each sample, trimmed reads were compiled into count tables of unique sequences, and samples were filtered for unique sequences of at least 20 nucleotides in length that were contained in all three EV-exposed *P. aeruginosa* samples but none of the control samples. The origin of 1,022 unique sequences matching these criteria was determined using BLAST+ (61), as described above, and included a separate alignment to -taxids 287 (*P. aeruginosa*). GenBank accessions returned by BLAST were annotated using the R package rentrez (62). For EV RNA sequences that were of human origin, we generated IntaRNA targeting predictions (63, 64) for *P. aeruginosa* genes, as described in *IntaRNA Target Predictions*.

Standard mRNA library preps were also constructed for these samples previously described (19) to assess the impact of EVs on *P. aeruginosa* gene expression. Total RNA was isolated with the miRNeasy Mini Kit (Qiagen, Germantown, MD; cat. no. 217004), including the on-column DNA digestion. RNA was ribosome-depleted before library prep. Sequencing libraries were prepared with an Illumina Stranded mRNA Prep kit (Illumina). One hundred and fifty basepair single-end reads were generated using an Illumina MiniSeq. Raw reads were trimmed using CLC Genomics Workbench software (Qiagen). Trimmed reads were aligned to the *Pseudomonas aeruginosa* PA14 reference genome using CLC Genomics Workbench. Raw data are available from GEO as GSE228919.

### IntaRNA Target Predictions

We used the IntaRNA algorithm (63, 64) to predict whether human sRNAs transferred to *P. aeruginosa* by EVs might regulate *P. aeruginosa* gene expression by targeting bacterial mRNAs. *P. aeruginosa* UCBPP-PA14 reference sequences and standard parameters were used to obtain energy scores as a measure of the likelihood of RNA-RNA interaction based on sequence similarity. The more negative the energy score, the higher the likelihood of interaction. Three top candidate RNA fragments derived from a lncRNA, a tRNA, and an rRNA were selected based on predicted targeting of MexHI-OpmD and abundance in *P. aeruginosa* following EV exposure. To assess whether targeting predictions extend beyond *P. aeruginosa* to other common lung pathogens, we ran IntaRNA to predict targeting of MexHI-OpmD orthologs by our top candidate RNA fragments in *Burkholderia cenocepacia* J2315, a multidrug-resistant CF clinical isolate, and *Staphylococcus aureus* COL, a methicillin-resistant *Staphylococcus aureus* clinical isolate.

### Statistical Analysis

Data were analyzed with Prism 8 for macOS (version 9.3.1, GraphPad, San Diego, CA) and the R software environment for statistical computing and graphics version 4.1.1 (65) using appropriate statistical methods, as indicated in the figure legends. Statistically significant differences between bacterial growth curves were determined with permutation tests using the compareGrowthCurves function from the R package statmod (version 1.4.36) (66, 67).

## RESULTS

### EVs Increase the Fluoroquinolone Sensitivity of *P. aeruginosa*

**Figure 1.**
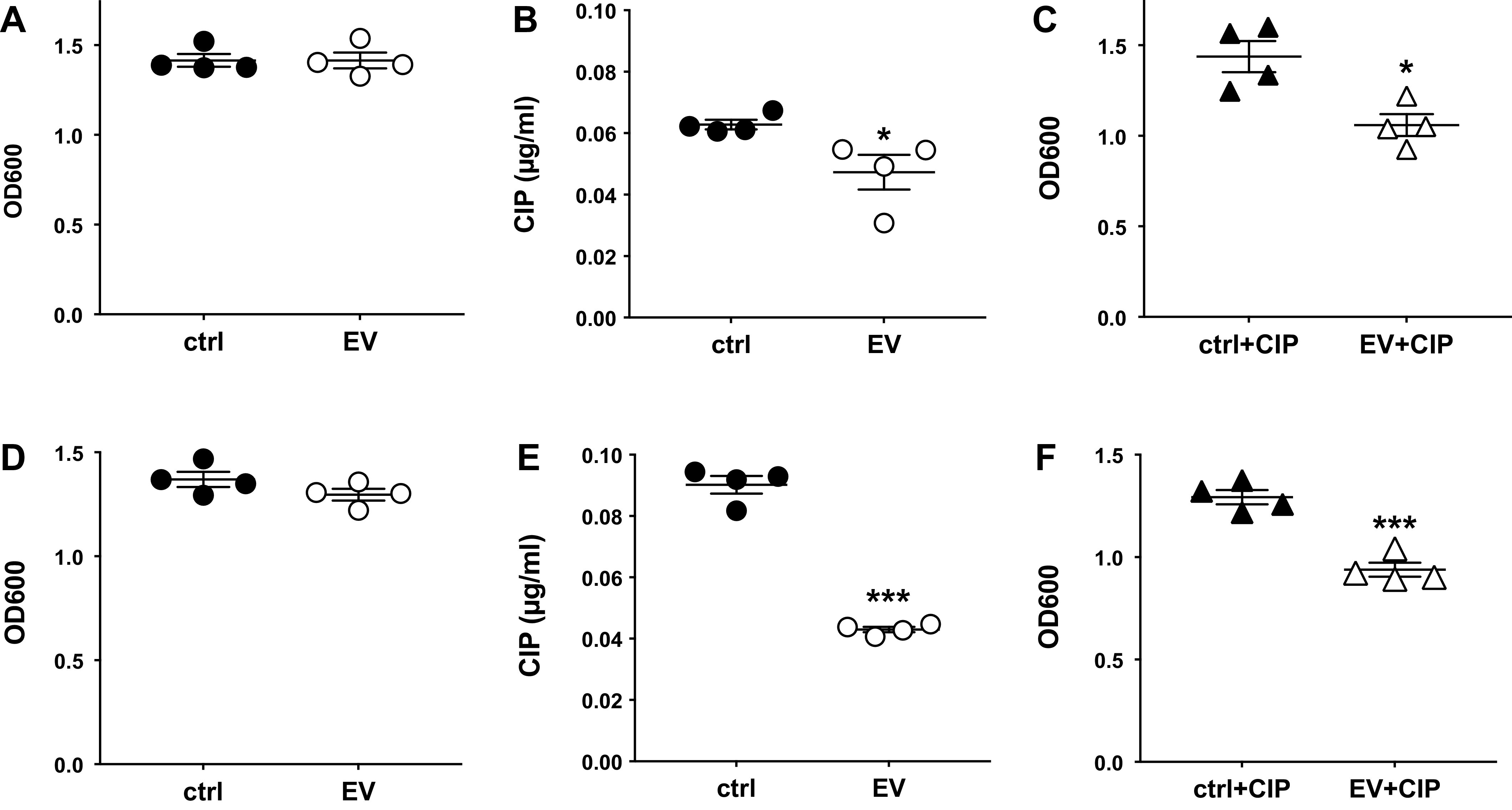
Extracellular vesicles (EVs) increase the fluoroquinolone sensitivity of *Pseudomonas aeruginosa*. EVs did not significantly affect planktonic growth [optical density at 600 nm (OD_600_)] of PA14 in the absence of ciprofloxacin (CIP) after a 20-hour incubation (*A*) but significantly reduced the minimum inhibitory concentration (MIC) of ciprofloxacin (*B*) as well as planktonic growth of PA14 in the presence of 0.02 µg/ml ciprofloxacin (*C*). EVs did not significantly affect planktonic growth (OD_600_) of the clinical isolate SMC1585 in the absence of ciprofloxacin after a 20-hour incubation (*D*) but significantly reduced the MIC of ciprofloxacin (*E*) as well as planktonic growth in the presence of 0.02 µg/ml ciprofloxacin (*F*). Statistical significance was determined using unpaired *t* tests. EVs were isolated from four AEC donors and each data point is the mean of 3 technical replicates from each donor. ***P < 0.001; *P < 0.05.

In a previous study, we demonstrated that EVs secreted by AEC increased the sensitivity of *P. aeruginosa* to beta-lactam antibiotics by delivering the miRNA let-7b to *P. aeruginosa* (19). To test the hypothesis that EVs may also alter the sensitivity to other antibiotics typically used to treat infections by *P. aeruginosa* (68), we examined the ability of EVs to alter the sensitivity of *P. aeruginosa* to the fluoroquinolone antibiotic ciprofloxacin. To that end, the planktonic growth of *P. aeruginosa* strain PA14 as well as the mucoid *P. aeruginosa* CF clinical isolate SMC1585 (50) was measured in the presence and absence of EVs and in the presence and absence of eight different concentrations of ciprofloxacin to determine the minimal inhibitory concentration (MIC). EVs alone had no significant effect on planktonic growth of PA14 in the absence of ciprofloxacin (Fig. 1*A*) but reduced the MIC of ciprofloxacin by 25% (Fig. 1*B*). EVs reduced planktonic growth of PA14 in the presence of 0.02 µg/ml ciprofloxacin (corresponding to about one-third of the MIC) by 26%, compared to 0.02 µg/ml ciprofloxacin alone (Fig. 1*C*). Likewise, EVs alone had no significant effect on planktonic growth of clinical isolate SMC1585 in the absence of ciprofloxacin (Fig. 1*D*) but reduced the MIC of ciprofloxacin by 48% (Fig. 1*E*). EVs reduced planktonic growth of SMC1585 in the presence of 0.02 µg/ml ciprofloxacin by 27%, compared to 0.02 µg/ml ciprofloxacin alone (Fig. 1*F*). Full 20-h growth curves for EV-exposed and vehicle exposed PA14 and SMC1585 in the presence and absence of 0.02 µg/ml ciprofloxacin are shown in Supplemental Fig. S1 (see https://doi.org/10.6084/m9.figshare.22302847.v1). In the presence of 0.02 µg/ml ciprofloxacin, there was a statistically significant difference in the growth curves of PA14 and SMC1585 with EVs compared to controls for the last 4 h of growth. Thus the combination of EVs and ciprofloxacin reduced the MIC of ciprofloxacin as well as the growth of PA14 and a clinical isolate of *Pseudomonas* (SMC1585). To assess whether the ExoQuick-TC EV isolation reagent affected *P. aeruginosa* antibiotic sensitivity, we conducted growth curve experiments with PA14 exposed to cell culture media not exposed to human bronchial epithelial cells and processed with ExoQuick-TC (EQ ctrl) as well as PA14 exposed to PBS vehicle control (ctrl), both in the presence of 0.02 µg/ml ciprofloxacin (CIP). There was no significant difference between the growth curves of PA14 exposed to PBS vehicle (ctrl + CIP) and PA14 exposed to the ExoQuick-TC control (EQ ctrl + CIP; Supplemental Fig. S1*C*). Thus the ExoQuick-TC EV precipitation reagent by itself did not significantly alter the sensitivity of PA14 to ciprofloxacin in the absence of EVs compared to PBS control.

### EVs Increase the Fluoroquinolone Sensitivity of *P. aeruginosa* by Targeting MexHI-OpmD

To begin to elucidate the mechanism whereby EVs increased the sensitivity of *P. aeruginosa* to ciprofloxacin, we analyzed the proteome of *P. aeruginosa* that had been exposed to EVs or vehicle control. EVs reduced the protein levels of all three subunits of the fluoroquinolone efflux pump MexHI-OpmD (Table 1). Compared to controls, EVs decreased protein levels of MexH by 48%, MexI by 50%, and OpmD by 35%. Normalized peak intensities for each sample set as well as average log_2_-fold changes and *P* values for all 1,619 detected proteins in *P. aeruginosa* are provided as Supplemental Table S1 (see https://doi.org/10.6084/m9.figshare.19701406.v1).

**Table 1.** EVs significantly reduce the protein levels of all three subunits of the fluoroquinolone efflux pump MexHI-OpmD

Uniprot ID	Locus	Name	Product	Log_2_ FC	%Decrease	*P* Value
A0A0H2ZGA2	PA14_09530	MexH	RND efflux membrane fusion protein	−0.93	−48	0.0008
A0A0H2ZGB3	PA14_09520	MexI	RND efflux transporter	−1.01	−50	0.0003
A0A0H2ZF64	PA14_09500	OpmD	Outer membrane protein	−0.62	−35	0.0161

Protein level changes in *Pseudomonas aeruginosa* exposed to extracellular vesicles (EVs) compared to vehicle controls; *n* = 3 biological replicates of EVs secreted by different airway epithelial cell donors. Statistical significance was determined with QPROT. FC, fold change; RND, resistance nodulation division.

**Figure 2.**
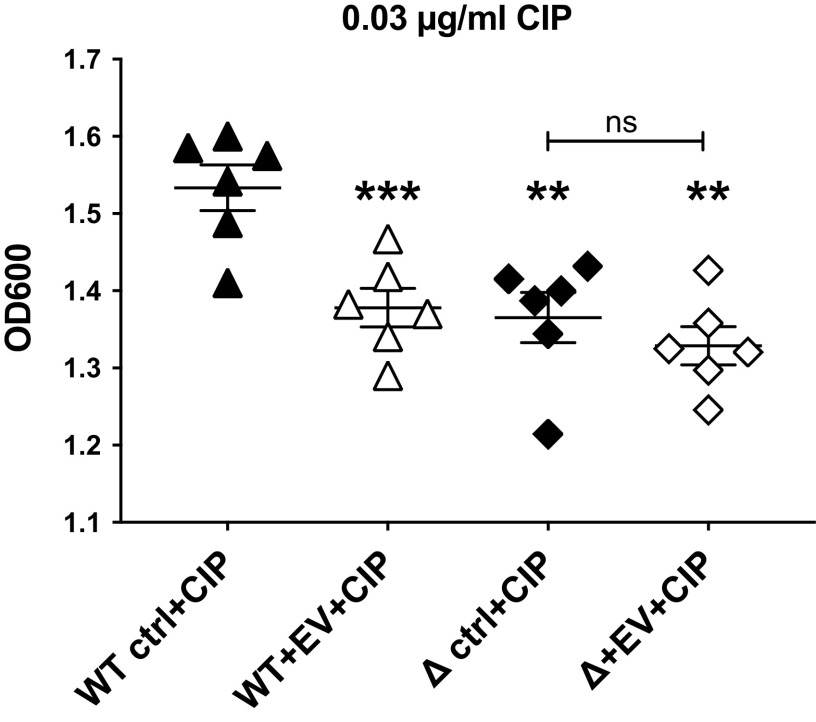
Extracellular vesicles (EVs) increase *Pseudomonas aeruginosa* ciprofloxacin sensitivity by targeting the fluoroquinolone efflux pump MexHI-OpmD. EVs decreased planktonic growth of wild-type (WT) PAO1 in the presence of ciprofloxacin (0.03 µg/ml). EVs in the presence of ciprofloxacin (CIP) (0.03 µg/ml) had no significant effect on the *mexHI-opmD* knockout strain (Δ + EV). Statistical significance was determined with repeated measures one-way ANOVA followed by Dunnett’s multiple comparisons test. EVs were isolated from 6 airway epithelial cell donors and each data point is the mean of 3 technical replicates from each donor. ***P < 0.001 compared to WT control (ctrl); **P < 0.01 compared to WT ctrl. OD_600_, optical density at 600 nm; ns, not significant.

To determine whether a reduction of MexHI-OpmD alone increased fluoroquinolone sensitivity, planktonic growth experiments were performed with a *mexGHI-opmD* deletion strain of *P. aeruginosa* PAO1 and the parental PAO1 wild-type strain. In the presence of ciprofloxacin EVs significantly decreased planktonic growth of wild-type PAO1 (Fig. 2). By contrast, in the presence of ciprofloxacin, EVs had no significant effect on the *mexGHI-opmD* deletion strain (Δ + EV). Supplemental Fig. S2 (see https://doi.org/10.6084/m9.figshare.19701337) contains full 20-h growth curves for the data shown in Fig. 2. Taken together, the data suggest that EVs increase *P. aeruginosa* ciprofloxacin sensitivity by targeting and downregulating the fluoroquinolone efflux pump MexHI-OpmD.

### Characterization of sRNA Content of EVs

We previously found that EVs secreted by AEC deliver the miRNA let-7b-5p to *P. aeruginosa*, where it reduced biofilm formation and increased sensitivity to beta-lactam antibiotics by targeting key genes involved in biofilm formation and beta-lactam resistance (19). However, let-7b-5p alone did not reduce protein levels of MexHI-OpmD. To determine whether a different sRNA was responsible for the EV-mediated reduction in MexHI-OpmD protein levels and the resulting increased sensitivity to fluoroquinolone antibiotics, we reanalyzed our existing RNA-seq data (GSE174690) to identify sRNAs in EVs that are predicted to target MexHI-OpmD. The alignment-based characterization of EV sRNA content showed that tRNA and tRNA-like fragments were the most abundant sequences in EVs, accounting for 65% of all aligned reads (Table 2). To gain a better understanding of the sRNA content of EVs at the sequence level, we generated count tables for unique sequences and filtered them for the most abundant unique sequences with at least a 20-nucleotide length and at least 100 counts in each sample. The resulting 1,346 sequences were aligned to homo sapiens using BLAST+ to obtain annotations for the 909 most abundant unique sequences with 100% identity and coverage (Supplemental Table S2; see https://doi.org/10.6084/m9.figshare.19701445.v1). Collectively, these 909 unique sequences accounted for 60% of the total reads detected in all 3 replicate samples. The sequences with the highest total count (listed at the top of Supplemental Table S2) include many tRNA and lncRNA fragments.

**Table 2.** Alignment-based characterization of EV sRNA content

RNA Type	%Total Reads
tRNA	46.0
tRNA like	19.0
RefSeq anti	16.5
RefSeq	9.9
rfam	3.0
rRNA	1.8
Other ncRNA	1.4
piRNA	1.1
lncRNA	0.7
miRNA	0.3
lncRNA anti	0.3
Other ncRNA anti	0.1
CDBox	0.003

RNA types (*column 1*) and their relative abundance as a percentage of total reads (*column 2*). Extracellular vesicles (EVs) were derived from 3 individual airway epithelial cell donors. tRNA, transfer RNA; RefSeq, messenger RNA; anti, antisense; rfam, noncoding RNA annotated in rfam database; rRNA, ribosomal RNA; ncRNA, noncoding RNA; piRNA, piwi-interacting RNA; lncRNA, long noncoding RNA; miRNA, microRNA; CDBox, small nucleolar RNA (snoRNA).

### Transfer of EV sRNAs to *P. aeruginosa*

We previously described the transfer of miRNAs secreted in AEC EVs to *P. aeruginosa* (19). To assess the transfer of other sRNAs besides miRNAs to *P. aeruginosa*, we reanalyzed RNA-seq data of EV-exposed *P. aeruginosa* and unexposed controls (GSE174710). We generated count tables of unique sequences from trimmed reads for individual samples: three unexposed controls and three samples of *P. aeruginosa* exposed to EVs secreted by different AEC donors. Reads were filtered to include only sequences with a length of at least 20 nucleotides that were detected in all 3 EV-exposed *P. aeruginosa* samples and none of the unexposed control samples. Filtering on these criteria yielded 1,022 sequences, 15 of which were perfect matches to human sequences in a BLAST search. The length requirement of at least 20 nucleotides was chosen because shorter sequences tend to be less specific and map to many different regions in the genome. The 15 human EV sRNA sequences listed in Table 3 were detected in EV-exposed *P. aeruginosa* but not in any of the unexposed control samples, suggesting that they are delivered to *P. aeruginosa* by EVs. Subsequently, we used IntaRNA to predict the target genes of these 15 human EV sRNAs in *P. aeruginosa.*

**Table 3.** Human EV RNA fragments delivered to P. aeruginosa

Sequence	nt	Description	Total Count	*mexH* Energy	*mexI* Energy	*opmD* Energy
TGGATTTTTGGAGCAGGGAG	20	CpG island DNA genomic Mse1 fragment	7	−13.19	−15.07	−17.44
**AAATGGATTTTTGGAGCAGGGAG**	**23**	**LHRI_LNC217 lncRNA**	**24**	−**13.18**	−**15.06**	−**17.43**
AAATGGATTTTTGGAGCAGGG	21	LHRI_LNC217 lncRNA	9	−8.68	−15.46	−16.97
**GCATTGGTGGTTCAGTGGTAGAATTCTCGCCTG**	**33**	**tRNA-Gly-GCC**	**11**	−**9.16**	−**14.77**	−**15.15**
**GCGGCGTCCGGTGAGCTCTCGCTGGCC**	**27**	**External transcribed spacer 18S ribosomal RNA**	**11**	−**10.52**	−**15.68**	−**14.77**
TGAGGTAGGAGGTTGTATAGTT	22	microRNA hsa-let-7e-5p (mature)	28	−10.01	−13.55	−14.6
TGAGGTAGTAGGTTGTATGGTT	22	microRNA let-7c-5p (mature)	21	−11.61	−13.67	−11.3
CAGGGATAACTGGCTTGTGGCGGCCAAG	28	External transcribed spacer 18S ribosomal RNA	4	−9.29	−13.95	−10.94
TCCCACATGGTCTAGCGGTTAGGATTCCTGG	31	tRNA-Glu-TTC	5	−10.26	−14.27	−10.92
AGTAAGGTCAGCTAAATAAGCTATCGGGCCCATACCCCGAAAATGTTGGTTATA	54	Mitochondrial tRNA-fMet	6	−8.18	−10.98	−8.97
AGTAAGGTCAGCTAAATAAGCTATCGGGCCCATACCCCGAAAATGTTGGTTA	52	Mitochondrial tRNA-fMet	8	−8.27	−10.96	−8.91
GAAAAAAAAAAAGGATGAGG	20	CKLF-like MARVEL transmembrane domain containing 6	7	−5.59	−9.04	−8.27
TGAGGTAGTAGATTGTATAGT	21	microRNA let-7f-5p, partial sequence (missing 3′ T)	26	−7.59	−10.77	−7.83
CGCGACCTCAGATCAGACGTGGCG	24	External transcribed spacer 18S ribosomal RNA	6	−3.93	−5.8	−3.2
TCCCTGAGACCCTTTAACCTGT	22	miR-125a-5p, partial sequence (missing 3′ GA)	11	−8.78	−8.7	0

Listed are 15 human extracellular vesicle (EV) RNAs that were exclusively detected in EV-exposed *Pseudomonas aeruginosa.* Three sequences that were top candidates for follow-up experiments based on abundance and targeting prediction for MexHI-OpmD are highlighted in bold font. *Column 1*: sRNA sequence; *column 2*: sequence length in nucleotides (nt); *column 3*: sequence identity as determined with BLAST+; *column 4*: total number of reads detected in *P. aeruginosa* after EV exposure; *columns 5*, *6*, and *7*: IntaRNA energy scores for *mexH*, *mexI*, and *opmD*, respectively. EVs were derived from 3 individual airway epithelial cell donors.

### Human EV sRNAs Are Predicted to Target *P. aeruginosa* Efflux Pump mexHI-opmD

To predict which *P. aeruginosa* genes the 15 human sRNAs that were delivered to *P. aeruginosa* by EVs may target, the RNA-RNA interaction tool IntaRNA (63, 64) was used to calculate binding energy scores. The more negative the energy score, the higher the likelihood of an RNA-RNA interaction. Table 3 lists the IntaRNA energy scores for each of the 15 EV sRNAs for predicted targeting of the fluoroquinolone efflux pump subunits *mexH*, *mexI*, and *opmD*. Three sRNAs with a high count in PA14 + EV samples and the best (i.e. lowest) energy scores for targeting of *mexH*, *mexI*, and *opmD* were selected for follow-up validation experiments: a 23-nt lncRNA fragment, a 33-nt tRNA-Gly fragment, and a 27-nt fragment of an 18S rRNA external transcribed spacer (highlighted in bold in Table 3). As described next, of the three top candidate sRNAs based on bioinformatic predictions, only the rRNA fragment could be validated in wet laboratory experiments to both increase the fluoroquinolone sensitivity of *P. aeruginosa* and to decrease MexHI-OpmD protein levels. The lncRNA fragment did not increase the fluoroquinolone sensitivity of *P. aeruginosa.* Although the tRNA fragment increased the fluoroquinolone sensitivity of *P. aeruginosa*, it did not reduce protein levels of MexHI-OpmD, suggesting that it acts through a different mechanism to reduce the fluoroquinolone sensitivity of *P. aeruginosa.*

### A Human rRNA Fragment Mediates the Increased Fluoroquinolone Sensitivity of *P. aeruginosa* by Targeting MexHI-OpmD

**Figure 3.**
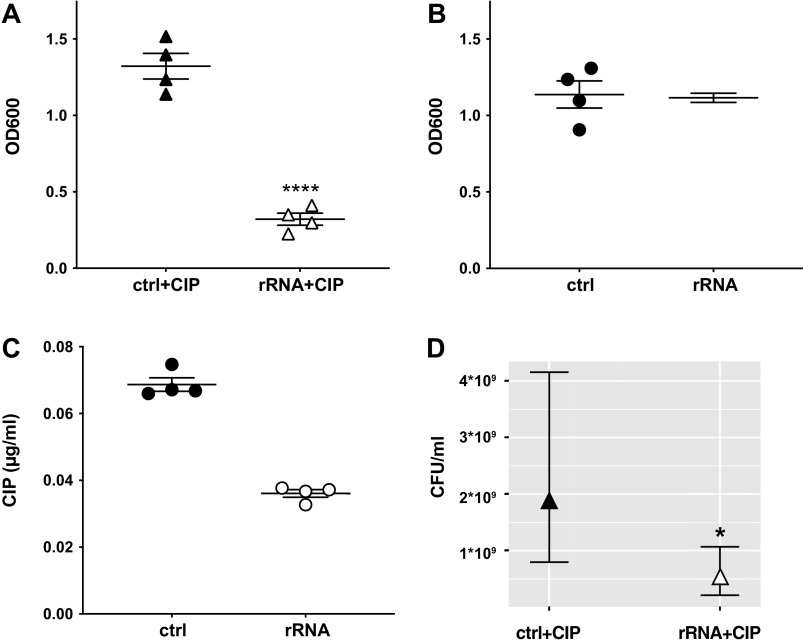
A human ribosomal RNA (rRNA) fragment increases the fluoroquinolone sensitivity of *Pseudomonas aeruginosa*. Compared to the empty vector control (ctrl), the rRNA fragment significantly reduced planktonic growth [optical density at 600 nm (OD_600_)] of PA14 in the presence of 0.03 µg/ml ciprofloxacin (CIP) and 100 mM arabinose after a 20-hour incubation (*A*) but did not significantly affect planktonic growth in the absence of ciprofloxacin (*B*). The rRNA fragment also significantly reduced the minimal inhibitory concentration of ciprofloxacin (*C*) as well as the number of live bacteria in the presence of 0.03 µg/ml ciprofloxacin (*D*). Statistical significance was determined using unpaired *t* tests for *A-C*; *n* = 4 independent experiments using different clones of each strain. Each data point is the mean of 3 technical replicates. A negative binomial regression model was used to analyze data in *D*; *n* = 3 independent experiments using different clones of each strain. *D*: batch-corrected estimates from the regression model. CFU, colony forming units. We used a negative binomial regression model to analyze the CFU/ml data, as previously published by us (69) and others (70), because it is a more powerful and less biased statistical model as it best fits bacterial counts, which are always greater than zero and skewed toward larger numbers. ****P < 0.0001 compared to ctrl; *P < 0.05 compared to ctrl.

To test whether the 18S rRNA spacer fragment in EVs mediates the increased ciprofloxacin sensitivity of *P. aeruginosa* in response to EVs, we expressed the rRNA fragment (sequence listed in Table 3) in *P. aeruginosa* using a plasmid with an arabinose-inducible promoter and measured planktonic growth in the presence and absence of ciprofloxacin using the empty vector as the control strain (ctrl). We observed that the rRNA fragment significantly decreased planktonic growth of *P. aeruginosa* in the presence of ciprofloxacin (0.03 µg/ml, Fig. 3*A*), while it did not significantly alter planktonic growth in the absence of ciprofloxacin (Fig. 3*B*).

Full 20-h growth curves for the data in Fig. 3, *A* and *B*, are provided in Supplemental Fig. S3 (see https://doi.org/10.6084/m9.figshare.19701352). The rRNA fragment also significantly decreased the MIC of *P. aeruginosa* for ciprofloxacin (Fig. 3*C*) and significantly reduced the number of live bacteria in the presence of ciprofloxacin (Fig. 3*D*). In the presence of the rRNA fragment, planktonic growth of *P. aeruginosa* was reduced by 76% compared to the empty vector control, the MIC of *P. aeruginosa* for ciprofloxacin was decreased twofold, and the number of live bacteria was reduced by 73%.

To validate that the rRNA fragment increased the ciprofloxacin sensitivity of *P. aeruginosa* by targeting the fluoroquinolone efflux pump MexHI-OpmD, we measured protein levels of MexHI-OpmD in the presence and absence of the rRNA fragment and found that compared to the empty vector control, the rRNA fragment decreased MexH by 53%, MexI by 26%, and OpmD by 52% (Table 4). The decrease in the protein levels of MexH and OpmD was statistically significant, and there was a trend for the rRNA fragment to decrease MexI protein levels that did not quite reach statistical significance. Normalized peak intensities for each sample set as well as average log_2_-fold changes and *P* values for all 1,911 detected proteins in *P. aeruginosa* are provided as Supplemental Table S3 (see https://doi.org/10.6084/m9.figshare.19701451.v1). Taken together, these experiments demonstrate that the rRNA fragment increased the ciprofloxacin sensitivity of *P. aeruginosa* by targeting the fluoroquinolone efflux pump MexHI-OpmD.

**Table 4.** A human rRNA fragment significantly reduces protein levels of MexH and OpmD

Uniprot ID	Locus	Name	Product	Log_2_ FC	%Decrease	*P* Value
A0A0H2ZGA2	PA14_09530	MexH	RND efflux membrane fusion protein	−1.09	−53	0.0003
A0A0H2ZGB3	PA14_09520	MexI	RND efflux transporter	−0.43	−26	0.1344
A0A0H2ZF64	PA14_09500	OpmD	Outer membrane protein	−1.07	−52	0.0107

Protein level changes in a *Pseudomonas aeruginosa* strain induced with 100 mM arabinose to express the ribosomal RNA (rRNA) fragment compared to the empty vector control strain; *n* = 3 different clones of each strain. Statistical significance was determined with QPROT. FC, fold change; RND, resistance nodulation division.

Finally, to begin to examine the mechanism whereby EVs reduce the protein levels of MexH, MexI, and OpmD, we analyzed RNA-seq data in which *P. aeruginosa* was exposed to EVs or control. Complete RNA-seq data are available (GSE228919). We found that mRNA levels of mexH increased by 35% (*P* = 0.0203), mexI mRNA increased by 54% (*P* = 0.0008), and opmD mRNA increased by 87% (*P* = 0.0001) in response to EV exposure, suggesting that the mechanism of action is not mRNA degradation but rather inhibition of translation, which is a well-known effect of miRNAs. These increases in mRNA may represent a positive feedback response to a reduction in MexH, MexI, and OpmD protein expression. Additional experiments, beyond the scope of the present study, are required to elucidate the mechanism whereby the rRNA reduces the fluoroquinolone efflux pump MexHI-OpmD.

## DISCUSSION

We report that AECs secrete EVs containing lncRNA, tRNA, and an 18S rRNA external transcribed spacer fragments that are transferred to *P. aeruginosa* and that EVs increased the fluoroquinolone sensitivity of *P. aeruginosa* by reducing protein levels of the drug efflux pump MexHI-OpmD. An 18S rRNA external transcribed spacer fragment by itself significantly reduced the protein levels of MexH and OpmD, thereby increasing antibiotic sensitivity to ciprofloxacin. The finding that AEC EVs contain rRNA fragments is consistent with prior characterizations of the sRNA content of EVs secreted by airway epithelial cells (40, 41). It has been previously described that rRNA fragments are produced in eukaryotes in a controlled manner that is population, sex, and tissue specific, suggesting they constitute another class of small noncoding regulatory RNAs (71, 72). Several reports predict and speculate about a regulatory function of rRNA-derived fragments, with some studies providing evidence for the regulation of various biological functions by rRNA fragments (71, 73, 74). Chen et al. (74) report RNAi knockdown of an endogenous 20-nt 28S rRNA fragment in the human nonsmall cell lung carcinoma cell line H1299 induced apoptosis and inhibited cell proliferation, while Wei et al. (75) found that endogenous rRNA fragments detected in human and murine liver regulate glucose metabolism in the murine hepatoma cell line Hepa 1–6 and are differentially expressed in the livers of diabetic mice. Moreover, several studies show coimmunoprecipitation of eukaryotic rRNA fragments with Argonaute proteins (75–77), further corroborating our conclusion that rRNA fragments are involved in the regulation of gene expression. Most of the previously described rRNA fragments are derived from 5.8S, 18S, or 28S rRNA as well as the internal transcribed spacer, and, in fact, some sequences that had been previously classified as miRNAs or piRNAs are in fact rRNA derived (78). There is very limited knowledge about the existence and function of fragments derived from the rRNA external transcribed spacer. Lee et al. (79) describe upregulation of rRNA external transcribed spacer fragments in *Neurospora crassa* in response to DNA damage, while Gupta et al. (80) found that fragments derived from an rRNA external transcribed spacer are upregulated in the parasitic protist *Entamoeba histolytica* in response to growth stress, suggesting that these fragments play a regulatory role in the stress response. However, direct evidence for the regulation of gene or protein expression by rRNA external transcribed spacer fragments has not been published. In addition, to our knowledge there are no studies showing cross-kingdom transfer of rRNA fragments from human EVs to a bacterial pathogen, nor is anything known about the effect of human rRNA fragments on bacterial gene expression. Thus, to our knowledge, our data represent the first report of an rRNA fragment secreted by a eukaryotic cell that regulates protein expression and alters the phenotype of a prokaryotic organism. Within prokaryotes, there are multiple mechanisms by which endogenous small RNAs regulate protein expression (81). In some cases, this regulation is mediated by Hfq, a *P. aeruginosa* protein known to facilitate the interaction of *P. aeruginosa* mRNAs with various endogenous regulatory small RNAs (82), yet in other cases binding and regulation are Hfq independent (83). However, nothing is known about the mechanism by which human sRNAs target prokaryotes to down-regulate protein expression. It is possible that the eukaryotic rRNA fragment may inhibit mexH, mexI, and opmD by binding to Hfq, but the rRNA could also act through an Hfq-independent mechanism. Additional studies beyond the scope of this work are needed to elucidate the mechanism whereby the human rRNA fragment reduces protein levels of MexH, MexI, and OpmD in *P. aeruginosa*.

We found that some, but not all, of the most abundant sequences in EVs can be detected in *P. aeruginosa* following EV exposure. However, some human EV sRNA sequences detected in EV-exposed *P. aeruginosa* are not among the most abundant EV sequences, suggesting differential stability in *P. aeruginosa*. While RNA contained within EVs is protected from degradation by extracellular RNases, once the EV RNA cargo is delivered to *P. aeruginosa*, RNA that is not protected by binding to protein or *P. aeruginosa* mRNA targets may be more prone to degradation, explaining the discrepancy between abundance in EVs compared to the abundance after delivery to *P. aeruginosa* for some of the sRNAs.

One limitation of our study is that we did not assess other AEC EV cargo such as DNA, protein, lipids, or metabolites, which could contribute to EV-induced sensitivity to ciprofloxacin. However, our experiment expressing the rRNA fragment in *P. aeruginosa* demonstrates that the rRNA fragment alone is sufficient to reduce the abundance of MexH and OpmD and to increase the sensitivity to ciprofloxacin.

Another limitation is that due to the redundancy of fluoroquinolone efflux pumps in *P. aeruginosa*, the suppression of MexHI-OpmD only leads to a partial effect on fluoroquinolone sensitivity. MexEF-OprN serves as another fluoroquinolone efflux pump in *P. aeruginosa* (51), and there are several additional efflux pumps that can serve this function (84), which helps explain why deletion of MexHI-OpmD does not result in a more drastic increase in fluoroquinolone sensitivity (Fig. 2). On the other hand, as shown in Fig. 2, the downregulation of MexHI-OpmD by EVs is enough to reduce fluoroquinolone sensitivity to a level achieved with a complete deletion of the efflux pump. Moreover, we found that expressing the rRNA fragment by itself led to a larger reduction in *P. aeruginosa* planktonic growth in the presence of ciprofloxacin than EVs and that while both EVs and the rRNA fragment alone led to a protein level reduction of the fluoroquinolone efflux pump MexHI-Opm, the rRNA fragment significantly suppressed additional proteins involved in mediating fluoroquinolone resistance, including MexA, GyrA, and ParC (84, 85). This may explain the increased ability of the rRNA fragment to reduce planktonic growth in the presence of ciprofloxacin compared to EVs, which contain additional cargo that may counteract the effect of the rRNA fragment.

*P. aeruginosa* in the lung is exposed to EVs secreted by other cell types in addition to those secreted by AEC, most notably immune cells like neutrophils and macrophages, whose EV RNA content may include the rRNA fragment in our study as well as other sRNAs that may affect antibiotic resistance of *Pseudomonas*. Therefore, a final limitation is that our study cannot elucidate the cumulative effect that EV sRNAs from multiple cell types may have on *P. aeruginosa* in the lungs.

Reanalysis of publicly available data (GSE126051) from a study characterizing sRNAs in human plasma (86) revealed that rRNA fragments identical in length and sequence to the rRNA fragment described in this study were present in plasma of healthy control subjects. Moreover, identical rRNA fragments were detected in EVs isolated from human bronchoalveolar lavage fluid (BALF; Dr. Alix Ashare, personal communication). These observations reveal the biological significance of our study since our rRNA fragment was found in clinical samples and is not limited to our model system of primary AECs. So far, there are no published studies that comprehensively characterize the sRNA content of EVs isolated from human BALF, and, as noted in a recent review by Zareba et al. (87), future in-depth RNA-seq profiling of distinct EV populations in BALF will elucidate their role in disease pathogenesis and provide insight into treatment strategies. The important questions of whether *P. aeruginosa* exposure alters the sRNA content of AEC EVs and whether there is a difference in the sRNA cargo of EVs secreted by WT and CF AEC need to be addressed in future studies.

To assess whether the rRNA targeting of efflux pumps is limited to *P. aeruginosa* or extends to efflux pump orthologs in other common lung pathogens we utilized IntaRNA to predict targeting of *Burkholderia cenocepacia* (which has orthologs for *mexH*, *mexI*, and *opmD*) and *Staphylococcus aureus* (which has an ortholog for *mexI*). Our candidate rRNA spacer fragment is predicted to target orthologs of *mexI* and *opmD* in *Burkholderia cenocepacia*, suggesting that AEC EV sRNAs are likely to affect the antibiotic sensitivity of other lung pathogens beyond *P. aeruginosa* and warrant future analysis beyond the scope of the current study.

In summary, our study provides the first evidence for transfer of lncRNA, tRNA, and rRNA fragments from eukaryotic EVs to a prokaryote and provides direct evidence for regulation of the prokaryote by a eukaryotic rRNA fragment, resulting in subsequent phenotypic alterations such as increased fluoroquinolone sensitivity. Importantly, our study examined EVs secreted by primary cultures of AECs obtained from three donors and the effects of the EVs on several strains of *P. aeruginosa*, including clinical strains, using biologically relevant concentrations of EVs. The development of new treatment approaches utilizing a combination of human rRNA fragments and antibiotics in nanoparticles or EVs may benefit individuals with chronic antibiotic-resistant *P. aeruginosa* infections.

## DATA AVAILABILITY

Data used in this report are openly available in the repositories noted in the text.

## SUPPLEMENTAL DATA

10.6084/m9.figshare.22302847.v1 Supplemental Fig. S1: https://doi.org/10.6084/m9.figshare.22302847.v1.

10.6084/m9.figshare.19701337 Supplemental Fig. S2: https://doi.org/10.6084/m9.figshare.19701337.

10.6084/m9.figshare.19701352 Supplemental Fig. S3: https://doi.org/10.6084/m9.figshare.19701352.

10.6084/m9.figshare.19701406.v1 Supplemental Table S1: https://doi.org/10.6084/m9.figshare.19701406.v1.

10.6084/m9.figshare.19701445.v1 Supplemental Table S2: https://doi.org/10.6084/m9.figshare.19701445.v1.

10.6084/m9.figshare.19701451.v1 Supplemental Table S3 https://doi.org/10.6084/m9.figshare.19701451.v1.

## GRANTS

This work was supported by funding from the Cystic Fibrosis Foundation (https://www.cff.org/) to B.A.S. (STANTO19G0, STANTO20P0, and STANTO19R0) and GREENE21G0 to D.A.H. and from the National Institutes of Health (https://www.nih.gov/) to B.A.S (P30DK117469 and R01HL151385) and to S.A.G. (R01GM122846).

## DISCLAIMERS

The funders had no role in study design, data collection and analysis, decision to publish, or preparation of the manuscript.

## DISCLOSURES

No conflicts of interest, financial or otherwise, are declared by the authors.

## AUTHOR CONTRIBUTIONS

K.K., T.H.H., D.A.H., and B.A.S. conceived and designed research; K.K., R.B., C.R., S.A.G., Y.A.G., B.-K.C., and D.M.V. performed experiments; K.K. and T.H.H. analyzed data; K.K. and T.H.H. interpreted results of experiments; K.K. prepared figures; K.K. drafted manuscript; K.K., T.H.H., S.A.G., Y.A.G., B.-K.C., D.A.H., and B.A.S. edited and revised manuscript; K.K., T.H.H., S.A.G., Y.A.G., B.-K.C., D.M.V., D.A.H., and B.A.S. approved final version of manuscript.
